# RetrogeneDB–a database of plant and animal retrocopies

**DOI:** 10.1093/database/bax038

**Published:** 2017-07-14

**Authors:** Wojciech Rosikiewicz, Michał Kabza, Jan G. Kosiński, Joanna Ciomborowska-Basheer, Magdalena R. Kubiak, Izabela Makałowska

**Affiliations:** a Department of Integrative Genomics, Institute of Anthropology, Faculty of Biology, Adam Mickiewicz University in Poznan, Umultowska 89, 61-614, Poznań, Poland

## Abstract

For a long time, retrocopies were considered ‘junk DNA’, but numerous studies have shown that retrocopies may gain functionality and become so-called retrogenes. Retrogenes may code fully functional proteins that coexist with parental gene products or may even replace them. Retrocopies may also function as regulatory RNAs and, for example, become a source of small interfering RNAs, act as *trans* natural antisense transcripts or as alternative targets for miRNAs. Numerous researchers have emphasized that retrogenes play a crucial role in various organisms’ developmental stages and diseases. Despite the ever-growing evidence of the importance of retrocopies, resources dedicated to retroposition are very limited. Here, we report an update of the RetrogeneDB, which, to the best of our knowledge, is the largest database dedicated to retrocopies. It provides annotations of 86 458 retrocopies in 62 animal and 37 plant species. The database contains information about the retrocopies’ localization, open reading frame conservation, expression, RNA Polymerase II activity and the alternative transcription start site studies. Orthologous relationships between retrogenes were also determined, which made retrocopy conservation studies much more valuable. Additionally, based on the RNA-Seq data from the Geuvadis project, the expression levels of retrocopies were estimated in a total of 50 individuals from 5 human populations. The information is now presented in a new, more user-friendly web interface, with easy access to the source data, which may be used for the downstream analysis. RetrogeneDB is freely available at http://yeti.amu.edu.pl/retrogenedb.

**Database URL:** http://yeti.amu.edu.pl/retrogenedb

**Secondary database URL:** http://rhesus.amu.edu.pl/retrogenedb

## Introduction

For a long time, retrocopies were considered ‘junk DNA’, a parental gene copy pasted randomly somewhere in the genome, bereft of all regulatory elements and chance for active transcription (1). Although, in principle, retrocopies are indeed ‘dead on arrival’, many of them gain functionality and again become a subject for selection (2). Retrocopies often code for fully functional proteins that coexist with the parental products (3) or even intercept the parental genes in their function after the latter, for whatever reason, become inactive (4). The results of numerous studies emphasize the key role of the expressed retrocopies, including regulatory functions as *trans* natural antisense transcripts (5–9), a source of small interfering RNAs (10, 11) or an alternative target for microRNAs originally aiming the parental gene transcripts (12–14). Expressed and functional retrocopies, called retrogenes, are today considered valuable players on the evolutionary playground (4, 9, 14–16).

RetrogeneDB was originally released in 2014 (17) and stands out from alternative databases such as RCPedia (18) or HOPPSIGEN (19) as a unique source of well-annotated retrocopies in 62 animal species. Here, we report an update of the RetrogeneDB database that now also includes retrocopies identified in 37 plant species, for a total of 99 studied species and 86 458 identified retrocopies. The RNA-Seq based expression level estimation of retrocopies and parental genes was updated and calculated for multiple tissues of 23 animal species. The expression of retrocopies was also validated based on the expressed sequence tags (ESTs) for 43 animal species. Additionally, for humans and mice, RNA Polymerase II activity regions and potential transcription start sites (TSS) were identified in the proximity of the 5’ ends of retrocopies. Finally, the expression of the selected human and mouse retrocopies was experimentally validated using standard PCR protocols. An orthologous relationship between retrogenes, in addition to the orthology of parental genes, was determined, thus making retrocopy conservation studies much more feasible. The information is now presented up in a new, more user-friendly web interface, with easy access to the source data, which may be used for downstream analysis. To the best of our knowledge, RetrogeneDB is now the largest and most complete existing repository of retrocopies of protein coding genes.

## Genome data and identification of retrocopies

**Figure 1.**
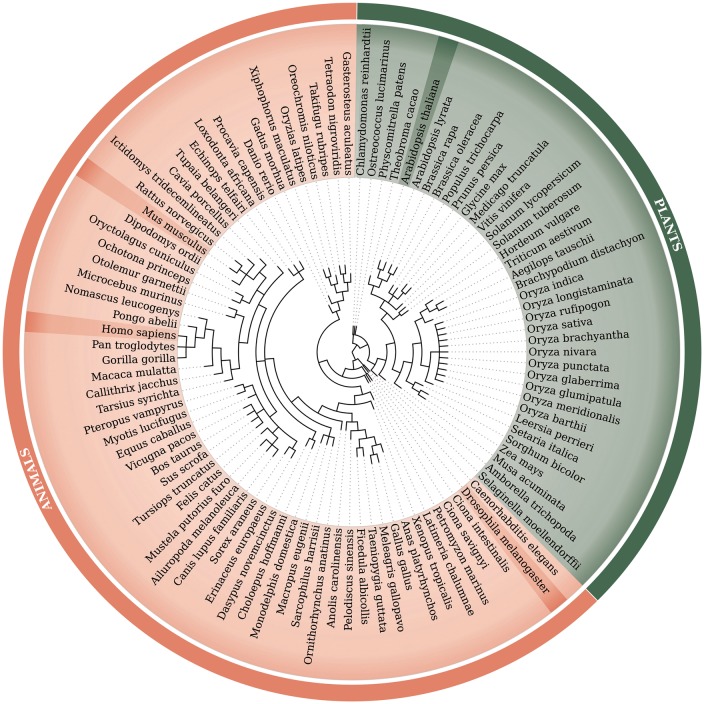
Phylogenetic tree of 99 species used within the updated RetrogeneDB database. The same tree is embedded into the database ‘browse’ web-page and interactively used to select a species of interest. Animal and plant species are colored in light red and green, respectively. Four chosen species, *Homo sapiens, Mus musculus, Drosophila melanogaster* and *Arabidopsis thaliana*, were highlighted for easier navigation. The web-based phylogenetic tree is drawn using the jsPhyloSVG library (37).

Retrocopies were identified for 99 *Eukaryotic* species, including 62 animal species [based on Ensembl Release 73 (20)] and 37 plant species [based on Ensembl Release 30 (21)]. The Ensembl database was a source of the known genes and transcript annotations and of genomic and proteomic sequences. The full list of species, with the genome assembly numbers, is included in Supplementary Material Table ST1. A phylogenetic tree of all analysed species, based on NCBI Taxonomy (22, 23), is represented in Figure 1.

Retrocopies were identified by searching for the sequence-based similarities between the hard-masked reference genomic sequences and protein sequences of all multi-exon, i.e having two or more exons, genes using LAST program (24), with an alignment length of at least 150 bp, and coverage and identity with parental protein of at least 50%. Additionally, a retrocopy was expected to show the loss of a minimum of two introns in comparison with the parental gene. If a newly annotated retrocopy had at least a 50% overlap with known pseudogenes from Ensembl annotations, its status was considered to be ‘KNOWN_PSEUDOGENE’. Otherwise, retrocopy was called ‘NOVEL’.

To identify annotated, protein-coding genes that originated via retroposition, all protein sequences of a given organism were aligned to themselves using LAST program (24). Next, alignments between different isoforms of the same gene were removed and genes whose coding sequence consisted of one exon in every transcript were selected. Each gene was then searched for the best-scoring alignment and examined for signs of retroposition. To call the gene a retrogene, the best alignment must had to have a coding sequence length at least 150 bp long, alignment identity and coverage at least 50% and the alignment had to span at least two parental gene’s introns, excluding the first and last 10 amino acids. Every retrogene found this way, was classified as ′KNOWN_PROTEIN_CODING′.

All parental genes with a large numbers of retrocopies assigned (>50 for mammals, 5 for other organisms) and an annotation ‘protein unknown’ were manually screened in order to minimize the number of false positive findings. Altogether 102 parental genes were checked and 47 were excluded, together with their retrocopies, due to ambiguous annotations of parental gene, consisting mostly of transposons, or vague conservation pattern. None of those were from well annotated genomes of model organisms.

To estimate the rate of possible false positives we compared identified human retrocopies with Ensembl database annotations. We choose human as our reference since this is the best annotated genome. Out of 4611 determined by us retrocopies only 121 were not annotated in Ensembl. Interestingly, 23 of them were later confirmed experimentally.

For more details about the protocols used to identify retrocopies and determine their expression levels, please see Supplementary Material file SM1.

## Retrocopies’ expression estimation

The expression of retrocopies was estimated using three main approaches: RNA-Seq data analysis, expressed sequence tags (ESTs) mapping to the reference genomes and, for selected retrocpies, by standard PCR method. For humans and mice, less direct methods of the expression validation using ChIP-Seq and CAGE (TSS) datasets were additionally applied.

## Expression estimation–RNA-seq

Estimating the expression of retrocopies based on RNA-Seq data is much more challenging than in the case of a standard RNA-Seq analysis. The difficulties are mostly related to the very high retrocopy-parental gene sequence similarity, which makes the distinction between the retrocopy and parental gene reads problematic or to a great extend impossible. Calculation of the expression level is even more difficult in the case of multiple retrocopies that arose from a single parental gene. Therefore, only uniquely mapped reads were considered to ensure that all of them truly originated from the particular retrocopy and not from its parental gene or ‘sister’ retrocopies. Such a strict approach resulted in the underestimation of the expression levels of both the parental gene and retrocopies. However, it reduced false-positive annotations of expressed retrocopies. Similarly conservative approach, i.e. considering only uniquely mapped reads, was applied in the RCPedia database (18). In comparison to the previous release of the database, the RNA-Seq expression level estimation, for all analysed species, was performed using a different set of programs, which resulted in the more accurate assignment of sequencing reads (see details in Supplementary Material file SM1).

The RNA-Seq-based estimation of the expression of retrocopies and parental genes was conducted for 145 libraries in 23 species that, together, possess 44 885 retrocopies. RNA-Seq libraries used in this analysis are listed in Supplementary Material Table ST2. Although at least one read was mapped uniquely to 63% of these retrocopies, the expression of 5776 of them was considered to be significant, i.e. equal to or higher than 1 RPM (reads per million mapped reads), which is the minimal expression level that we adopted to count a particular retrocopy as expressed [based on our previous studies (17)]. This slightly differs from the RCPedia, where as expressed were counted all retrocopies with minimum of five matching reads regardless of the library size (18).

The estimation of the expression level of human retrocopies was, in addition, conducted using RNA-Seq data of 50 individuals from 5 different human populations, generated within the Geuvadis RNA sequencing project (25) and listed in Supplementary Material Table ST3.

## Expression validation using EST

The expression of retrocopies was also validated using the expressed sequence tag (EST) sequences. ESTs for 43 species, listed in Supplementary Material Table ST4, were downloaded via UCSC Table Browser (26) and mapped to reference genomes using BLAT (27). A retrocopy was considered to be expressed if its alignment, with at least one EST, was a minimum of 100 nt long with at least 90% identity. Required identity level is relatively low, nevertheless we choose it due to possible sequencing errors, reverse transcriptase errors and variation. In addition however, the EST alignment to the retrogene had to be better, i.e. have a higher score and identity, than the alignment to any other sequence, including the parental gene.

EST data provided expression evidence for 557 retrocopies from 31 species. Most of these retrocopies were human (199 retrocopies) and mouse (113 retrocopies), which can be explained by the fact that the EST datasets were considerably larger for these two species. The expression of 293 retrocopies from 15 species was confirmed by both RNA-Seq and EST data.

The overall number of ESTs matching retrocopies (3106 sequences in total, Supplementary Material Table ST4) is relatively low. Two major factors could possibly contribute to this. First, retrocopies are known to have a very low expression and often limited to one or few tissues. Therefore, their transcripts may be not captured in low or moderate throughput experiments from restricted number of tissues. Secondly, sequences that matched equally well to a given retrogene progenitor were excluded what additionally reduced the number of positive results.

## Expression validation by PCR experiments

Experimental validation of retrocopies using standard PCR is often very difficult due to the very high sequence similarity between the parental genes and their retrocopies as well as the possibility of multiple retrocopies of the same parental gene. Therefore, it is usually very hard to design primers that will lead to unique product amplification. However, after a laborious work, the expression of 51 human and 11 mouse retrocopies was successfully confirmed experimentally. The applied experimental PCR protocols together with the electrophoresis gels are provided within the detailed view of each validated retrocopy. The selection of candidates for the experimental validation was determined by ongoing research on retrocopies performed in our laboratory and the feasibility of the primers’ design.

## ChIP-seq and CAGE data analysis

Additional support for the expression of human and mouse retrocopies is provided by ChIP-Seq RNA Polymerase II activity and CAGE transcription start sites data from the ENCODE (28) and FANTOM5 (29) projects, respectively. Taking advantage of these efforts we downloaded precomputed RNA Polymerase II Subunit A activity peaks and alternative transcription start sites (TSS) locations. Polymerase activity and/or TSS annotations were searched near retrocopy 5’ ends, that is, up to 1000 bp and 500 bp upstream of the annotated retrocopy 5’ end for RNA Polymerase II activity peaks and TSS annotations, respectively, and down to 30% of the annotated retrocopy body. It led to the identification of 233 human and 140 mouse retrocopies with the RNA Polymerase II activity signal and of 799 human and 796 mouse retrocopies with at least one TSS. Of these retrocopies, 89 human and 58 mouse retrocopies were associated with signals of both the RNA Polymerase II activity and TSS.

The database contains information about ChIP-Seq peaks from 53 human and 4 mouse libraries. However, for visualization in the web browser, all of the data were merged into two ChIP-Seq tracks by using the merge tool from BEDTools (30) and bigWigMerge from UCSC (31) for peaks and raw ChIP-Seq signals, respectively.

## Retrocopies orthology

The retroposition of the same parental gene may occur many times and independently in various species. Therefore, a similarity analysis is not sufficient to identify orthologous retrocopies. For that reason, to determine orthologous retrocopies, whole genome alignments from Ensembl were utilized. Blocks of alignment containing retrocopies were extracted from the whole genome alignments. Retrocopy orthologous groups were then constructed based on their overlap within alignment blocks, which required at least 50% reciprocal overlap (32). The analysis was performed in three sets: 13 *eutheria* species (*Homo sapiens, Pan troglodytes, Pongo abelii, Gorilla gorilla, Macaca mulatta, Callithrix jacchus, Mus musculus, Rattus norvegicus, Equus caballus, Sus scrofa, Bos taurus, Canis familiaris*, and *Oryctolagus cuniculus*), 5 *teleost* fish (*Tetraodon nigroviridis, Gasterosteus aculeatus, Takifugu rubripes, Oryzias latipes*, and *Danio rerio*) and 3 *neognath* birds (*Gallus gallus, Taeniopygia guttata*, and *Meleagris gallopavo*) (Supplementary Material Figure SF1). As a result, 4216, 14 and 10 orthologous groups were identified, respectively, containing 12 942 retrocopies in total.

## RetrogeneDB database composition and usage

**Figure 2.**
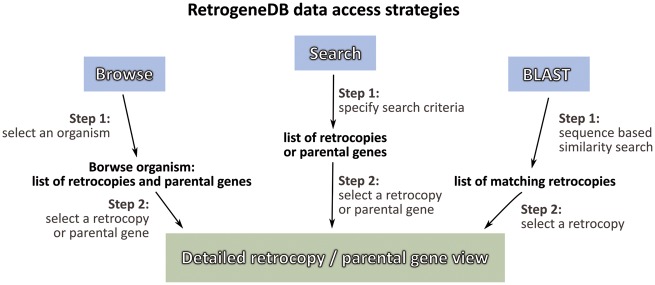
RetrogeneDB data access strategies.

Information about a specific retrocopy or its parental gene can be reached using one of the two main approaches: the Browse or Search functions, which are both located in the top menu panel of the page. Alternatively, a user may search our database based on a nucleotide sequence using the BLAST program (Figure 2).

### I. Browse

By selecting the ‘Browse’ option from the main menu, a user is provided with four ways of choosing the species or retrocopies of interest:

1. Browse using the species tree–a user may select one of the 99 species analysed within the RetrogeneDB database simply by clicking on its scientific name on the phylogenetic tree (Figure 1).
2. Browse animal species–a user may select one of the 62 animal species from the table listing common and Latin names together with a basic summary. The summary includes the total number of retrocopies identified for the organism and the number of retrocopies with a conserved open reading frame in column ‘ORF’, number of retrogenes with the expression signal from RNA-Seq, EST, ChiP-Seq, TSS as well as experimentaly confirmed using PCR method. In the case of human, the table provides also the number of retrocopies associated with the population specific indels, i.e. retroduplication variation (RDV), based on previous studies (32).
3. Browse the plant species tab–a user may select one of the 37 analysed plant species. For each species, the common and scientific names, number of identified retrocopies and number of retrocopies with conserved open reading frames are provided.
4. Browse the retrogene orthology tab–this allows users to select and list orthologous retrocopies that originated in a common ancestor genome.

### II. Species summary

**Figure 3.**
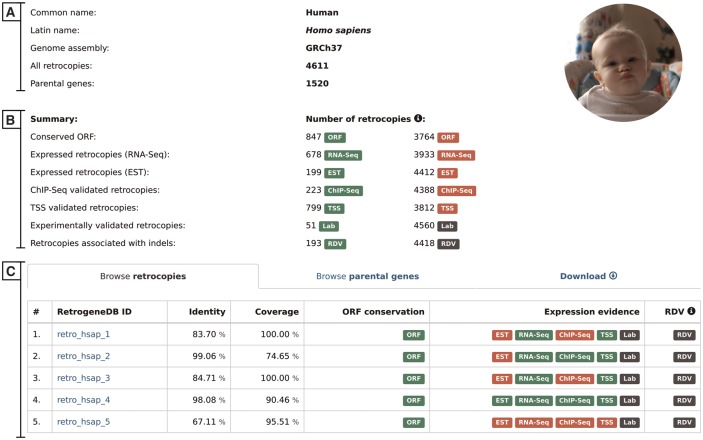
Selected species summary. This page provides a list of all retrocopies and parental genes together with access to the data download section. It also displays, whenever applicable, an overall view of the expression data. The page contains the following sections: (**A**) Basic information; (**B**) Summary, which lists the number of retrocopies supported by the additional data indicated by badge; and (**C**) Browse retrocopies, parental genes and download tabs.

The selection of a scientific or common species name from the browse pages leads the user to the species summary page. This page provides a list of all retrocopies and parental genes, and access to the data download section. It also displays, whenever applicable, an overall view of the expression data. The page contains the following:

1. Basic information and summary data–this section displays common and scientific names, the number of the Ensembl genome assembly used for the analysis, the total number of retrocopies and their parental genes for a given species, the number of retrocopies that possess conserved ORF and the number of retrocopies with evidence of expression or linked to population-specific indels (Figure 3A). A particular type of information is displayed only if it is applicable for the selected species.
2. Browse retrocopies tab–this gives a list of all retrocopies of a given species, together with basic information on the retrocopy, such as the parental gene alignment identity and coverage, ORF conservation, expression and, in the case of humans, evidence for RDV. For enhanced data visualization, each type of information is marked with a colored badge (Figure 3B). In the case of the expression data, a green badge represents the presence of evidence from a given data type, whereas its absence is marked in red. Grey badges are used for retrocopies with no evidence of RDV and for those for which expression validation, using standard PCR, was not conducted. To access the detailed description of the selected retrocopy, one must click its RetrogeneDB ID. The following page is described in the ‘detailed view of retrocopies’ section.
3. Browse parental genes tab–this gives a list of all parental genes, their Ensembl ID and alternative gene name, together with a short gene description provided by the Ensembl 73 and Ensembl Plants 30 annotations.
4. Download tab–by using this tab, a user may download, for the selected species, all retrocopies or parental genes in FASTA or BED format. Additionally, by choosing TSV format, a user may fully customize the output content and select required columns from the MySQL tables.

### III. Search

**Figure 4.**
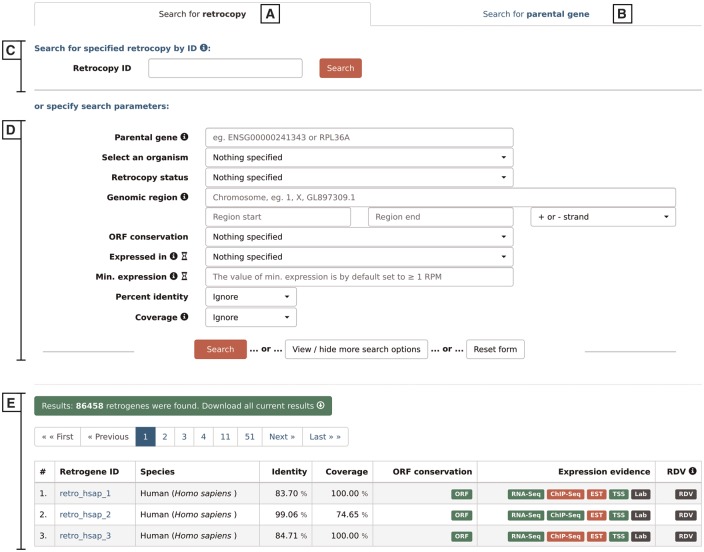
Search form that allows the user to search for either retrocopies (**A**) or parental genes (**B**), using Retrocopy ID or Ensembl accession number (**C**), or specifying various search parameters (**D**). The results are displayed at the bottom of the page (**E**).

By choosing the ‘Search’ option from the main menu, a user may search for either retrocopies (Figure 4A) or parental genes (Figure 4B). Both options allow the following:

1. Search by ID–(Figure 4C)–depending on the tab selected, the search field enables a search for a specific retrocopy (by typing the full retrocopy RetrogeneDB id or Ensembl accession number) or parental gene (by typing the parental gene name or full Ensembl accession number).
2. Search by specified parameters–(Figure 4D)–this section allows specific search parameters, including species, genomic regions, RNA-Seq or EST expression validation, minimal expression value and many more. In the case of a retrocopy search, not all options are displayed by default, but they can be viewed by clicking on the ‘View/hide more search options’ button. The search fields that may not be intuitive are accompanied by the small information ‘i' icon, which triggers a tooltip with an appropriate description. Multiple search parameters may be specified; however, with some of them, the search may be slower than a standard query. Thus, the title of each of the search fields that may potentially slow the search process is accompanied by an hourglass icon with an appropriate description in the tooltip.

After the ‘search’ button is activated, 50 results per page will be displayed (Figure 4E). One may navigate through the result pages by using navigation buttons or may download all of the currently displayed results. The output file for the results is a tab separated file (TSV), where the initial lines show the list of all search criteria that were changed from the default values and the column descriptions.

### IV. Blast

The ‘BLAST’ option allows a user to use a nucleotide or protein sequence in FASTA format to conduct a sequence-based similarity search using a BLAST algorithm. A user may specify the species or the taxonomical group against which the BLAST search will be performed. One may also specify the expected value and maximum number of results. In addition, an algorithm optimized either for medium or high sequence similarity may be selected by choosing BLASTN version 2.4.0+ or MEGABLAST version 2.2.26 (33), respectively. In the case in which a protein sequence is used as a query, a user should select the TBLASTN version 2.4.0+ algorithm (34). All other parameters remain as defaults and cannot be changed by the user.

### V. Detailed view of retrocopies

**Figure 5.**
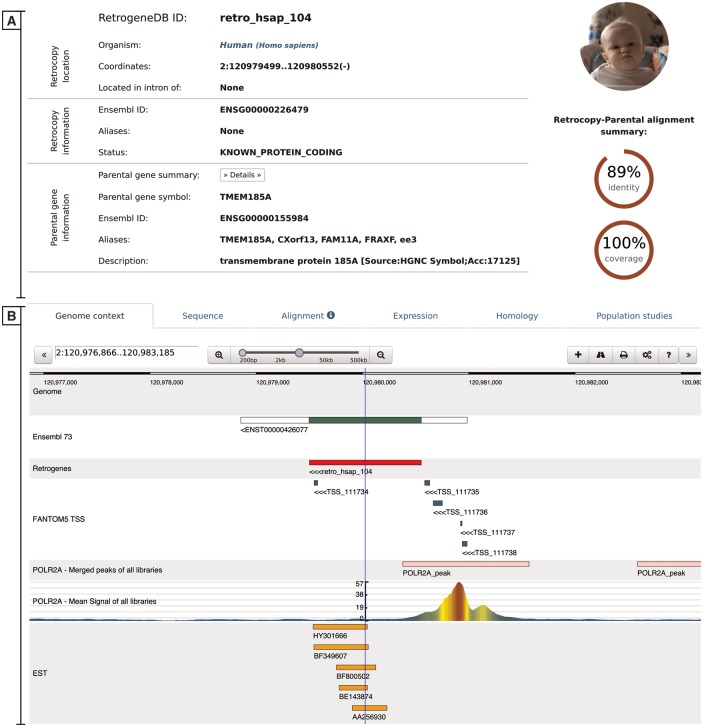
Retrocopy detailed view page comprising two basic sections: retrocopy summary (**A**), and the detailed information (**B**) contained within the following tabs: genome context; sequence of the retrocopy; retrocopy–parental gene protein alignment used for retrocopy identification; expression validation of the retrocopy; retrocopy and/or parental gene homology information; and in the case of humans, a population studies tab, which inform of retroduplication variation (RDV) evidence and retrocopy expression levels in 50 human libraries from 5 populations.

The retrocopy view page comprises two basic sections: the retrocopy summary and the detailed information contained within multiple tabs (Figure 5). The retrocopy summary provides the information on the retrocopy genomic coordinates, its status, its Ensembl accession number and alternative names, whenever available. In the retrocopy summary section, basic information about the parental gene is also provided, of which a more detailed view may be displayed by clicking the ‘Details’ button. Additional retrocopy information is displayed within multiple tabs as follows:

1. Genome context–this tab uses a dalliance web-browser (35) to allow the manual inspection of the genomic location of the retrocopy. Depending on data availability, up to 7 tracks can be displayed: reference genome, Ensembl annotations, retrocopy, TSS from the FANTOM5 project, aligned EST sequences and two ChIP-Seq tracks–RNA Polymerase II peaks and raw signals.
2. Sequence–this tab provides a retrocopy nucleotide sequence.
3. Alignment–this tab displays the alignment of the parental gene protein and retrocopy translation generated within the retrocopy annotation process. It contains information on frameshifts and of the stop codons incorporated into the retrocopy sequence, with respect to the parental gene’s protein sequence.
4. Expression–this tab provides information on the estimation of the retrocopy expression based on the given data type. If applicable, details about the PCR experiment, including designed primers and electrophoresis gel photos of the experimental validation of the retrocopies, are displayed. All of this information may be viewed by clicking on the appropriate badge.
5. Homology–this tab contains two sections. The first section displays orthologs of the retrogene, and the second section displays homologs of the parental gene. All species names, parental gene IDs and retrocopy IDs are linked to the corresponding detailed views.
6. Population studies–this tab is presented only for human data and shows the expression levels estimated based on the RNA-Seq fibroblast libraries of 50 individuals from 5 populations. A user may display the detailed expression values, which are represented visually on the map, by clicking the red ‘Show/hide detailed expression values’ button. Additionally, below the expression data, maps with RDVs frequencies within the selected populations are provided whenever applicable.

### VI. Parental gene detailed view

A detailed view of parental genes provides a summary of the parental genes’ Ensembl annotations, alternative gene names, genomic coordinates and gene descriptions. It also displays the genome browser, a canonical sequence used for the identification of retrocopies, the RNA-Seq based expression levels, the list of all retrocopies of this particular parental gene and the list of homologous parental genes and their retrocopies.

## Conclusions

This updated RetrogeneDB provides well-annotated information on the 86 458 retrocopies of 99 species, including 37 plant species that were not included in the previous version of the database. Retrocopies were annotated based on a very rigorous analysis, followed by a manual inspection. A detailed examination of retrocopies was performed to identify those with conserved ORFs and/or signals of transcriptional activity. For numerous species, the expression of retrocopies was estimated based on different types of data. Altogether, 195 RNA-Seq libraries for 23 species and EST data for 43 species were analysed. In addition, for humans and mice, an analysis of 57 ChIP-Seq libraries and 2901 CAGE experiments was performed. The expression of the selected 62 retrocopies was also experimentally validated in our laboratory by using the standard PCR protocols. Considering all of the applied methods, at least one expression signal was found for as many as 1322 human and 1100 mouse retrocopies. This indicates that even 30% of the retrocopies may possess some transcriptional potential and potentially represent retrogenes although; expression itself is not sufficient to consider a retrocopy as functional. In monotremes and non-mammalian species, this percentage may be much higher. In chickens, for example, 58% of the retrocopies were found to be transcriptionally active based on RNA-Seq and/or EST data. Interestingly, 40–70% of expressed retrocopies in placentals and marsupials have interrupted ORF; therefore, these copies, if they are truly functional, do not code for proteins, or at least not the same proteins as their parental genes. These retrocopies are good candidates for regulatory RNA genes.

In addition to ORF conservation and expression data, RetrogeneDB contains information about homologous parental genes in other species and retrocopy orthologs. Moreover, for each retrocopy, a genome browser is provided, thus allowing users to examine the retrocopy and related data in a broader genomic context. Taken together, RetrogeneDB is the most comprehensive information resource related to retrocopies. This applies to the number of analysed species and the utilized data sources.

## Future perspectives

In the next update of RetrogeneDB, we plan to incorporate histone modification studies from large-scale projects, such as the Encode Project, strengthened by an analysis of the possible transcription factor binding sites, using resources such as JASPAR (36), together with the supportive analysis of these binding sites utilizing ChIP-Seq data. With the growing amount of various sequence data, we also hope to provide stronger evidence of expression, particularly for species other than humans and mice. For individual retrocopies, we also plan to include additional literature support, with a description of the key aspects and the context of the individual research.

## Availability

The RetrogeneDB database is freely accessible via http://yeti.amu.edu.pl/retrogenedb. The secondary link to the database, located on a different server, may be accessed from http://rhesus.amu.edu.pl/retrogenedb. Additionally, the first, unchanged version of the database is accessible under http://retrogenedb.amu.edu.pl.

## Supplementary data

Supplementary data are available at *Database* Online.

Supplementary Material **Figure SF1.** Phylogenetic tree of 13 eutherians, 3 neognaths and 5 teleosts, for which the orthology of retrocopies was determined.

Supplementary Material **Figure SF2.** Visualization of the range in which transcription start sites – *TSS Region*, and RNA Polymerase II Subunit 2A ChIP-Seq peaks – *POLR2A Region*, are expected to be considered as potentially associated with the retrocopy.

Supplementary Material **Table ST1.** List of species and the used genome assemblies.

Supplementary Material **Table ST2.** List of RNA-Seq project accession numbers associated with organs among 23 species studied.

Supplementary Material **Table ST3.** List of 50 personal identifiers from the Geuvadis RNA sequencing project (25) that were used to download the RNA-Seq raw reads from fibroblasts and further used to estimate the expression level of retrocopies. Source population are as follows: CEU – Utah Residents (CEPH) with Northern and Western European Ancestry, FIN – Finnish in Finland, GBR – British in England and Scotland, YRI – Yoruba in Ibadan, Nigeria.

Supplementary Material **Table ST4.** List of 43 species which were analysed using EST data as well as the total number of retrocopies validated and the total number of ESTs mapped for these retrocopies for each species.

Supplementary Material **Table ST5.** ChIP-Seq libraries used for the association of RNA Polymerase II Subunit 2A peaks with retrocopies. The table contains a list of 53 human and 4 mouse ChIP-Seq peak files precomputed by The Encode Consortium, which were directly used for the association of RNA Polymerase II Subunit 2A peaks with retrocopies. The table also lists 76 human and 4 mouse corresponding ChIP-Seq raw signals, which were merged into one bigWig file per species and are visualized in the genomic browser of the detailed retrocopy view.

Supplementary Material **Table ST6.** Description of 1829 human and 1072 mouse CAGE experiments, of which the results were used for the association of retrocopies with the alternative transcription start sites.

## Funding

This work was supported by the National Science Centre in Poland (grants No. 2013/11/B/NZ2/02598 to I.M. and 2013/09/N/NZ2/01221 to M.K.) and Polish Ministry of Science and Higher Education (grant No. IP2011007571 to J.C.). Funding for open access charge: KNOW Poznan RNA Centre (grant No. 01/KNOW2/2014).

*Conflict of interest*. None declared.

## Supplementary Material

Supplementary Data
