# Supplementary figures and images for: RetrogeneDB–a database of plant and animal retrocopies
Source: Database (Oxford). 2017 Jul 14;2017:bax038. doi: 10.1093/database/bax038 (PMC5509963; doi:10.1093/database/bax038)

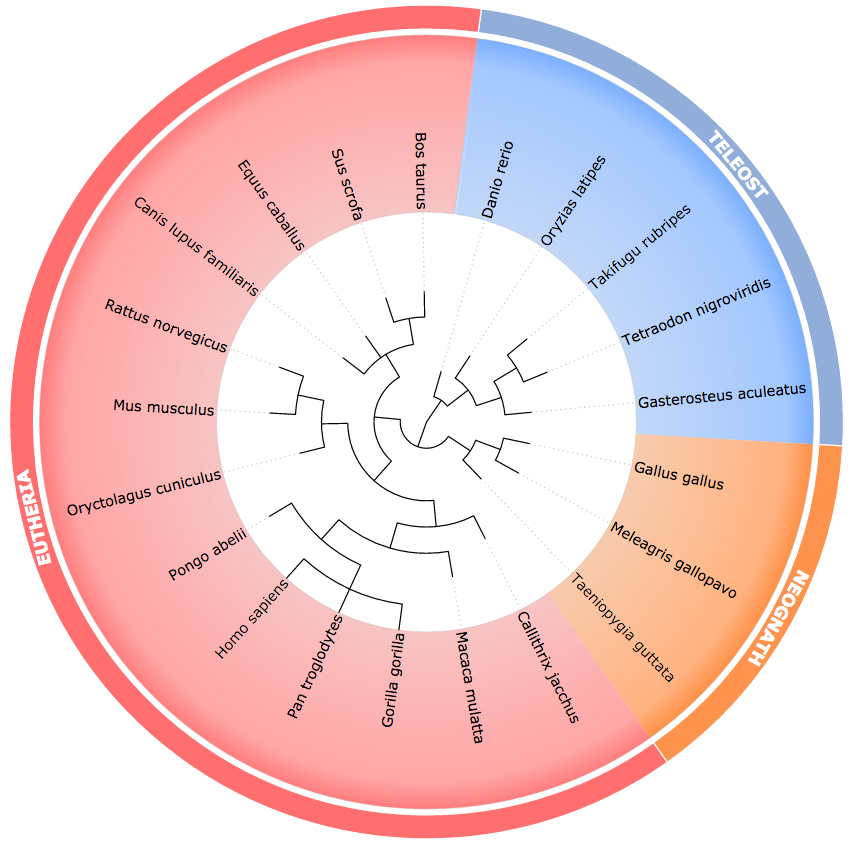

Supplement: Supplementary Data [file bax038_Supp.zip › SF1.png]

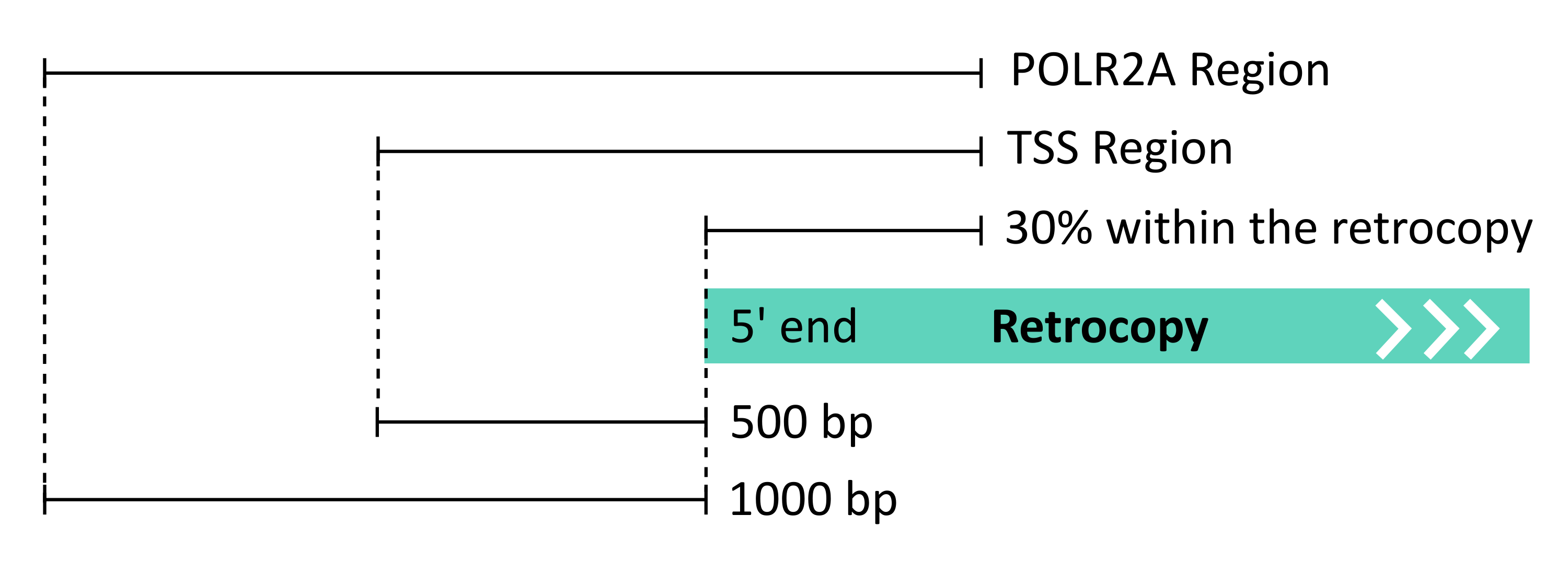

Supplement: Supplementary Data [file bax038_Supp.zip › SF2.png]
