# Supplementary material for: RetrogeneDB–a database of plant and animal retrocopies
Source: Database (Oxford). 2017 Jul 14;2017:bax038. doi: 10.1093/database/bax038 (PMC5509963; doi:10.1093/database/bax038)
Supplement: Supplementary Data [file bax038_Supp.zip › SM1.doc]

**Retrocopies identification**

Retrocopies were identified using the LAST program [1] by the translated protein sequence alignment to the hard-masked reference genome sequence. All sequences of all species were downloaded from Ensembl 73 [2] and Ensembl Plants 30 [3]. Species names with the genome assembly numbers are listed in supplementary table ST1. Genes that contain a reverse transcriptase domain were excluded from the set. The following parameters of the LAST program were applied:

- Substitution matrix: BLOSUM62
- Gap existence penalty: 11
- Gap extension penalty: 2
- Frameshift penalty: 15
- Drop-off value: 20

Multiple alignment hits to the same genomic *locus* were clustered using BEDTools [4]. A minimum overlap of 150 bp between alignments on the same strands was required to join them into a cluster. If a particular cluster overlapped a known protein-coding gene, it was excluded from further analysis. All remaining clusters were considered to be potential retrocopies. For each of these clusters, the alignment with the highest score was selected as optimal. Suboptimal alignments were selected if their score was at least 98% of the optimal alignment. A cluster was considered to be a retrocopy if the following criteria were fulfilled:

- Optimal or suboptimal alignment involved a loss of at least 2 introns, longer than 75 nt, in comparison to the parental gene (based on [5]);
- Alignment length was at least 150 bp long at the nucleotide level;
- Protein sequence-based identity and coverage were at least at the level of 50% at the protein level.

The final step included annotation of the parental gene identity and genomic coordinates of the retrocopy based on the best alignment from the selected cluster. In the case of two or more alignments that were equally good, the final alignment was chosen randomly. If a newly annotated retrocopy had at least a 50% overlap with known pseudogenes from Ensembl annotations, its status was considered to be “KNOWN_PSEUDOGENE”. Otherwise, retrocopy status was considered as “NOVEL”.

To identify annotated, protein-coding genes that originated via retroposition, all protein sequences of a given organism were aligned to themselves using LAST [1]. Proteome sequences were limited to the products of genes annotated in Ensembl as ‘protein-coding’. Alignments were subsequently filtered, and those between isoforms of the same gene were removed. Next, genes whose coding sequence consisted of one exon in every transcript were selected. If the gene encoded more than one protein, the longest isoform was chosen. Each gene was then searched for the best-scoring alignment and examined for signs of retroposition. To call the gene a retrogene, the best alignment must have met following criteria: neither tested gene nor its potential parental gene should show reverse transcriptase activity (detected from the Ensembl descriptions of protein families), the coding sequence of the tested gene is at least 150 bp long, the alignment identity and coverage of both proteins is greater than 50% and the aligned fragment with potential parental gene (excluding the first and last 10 amino acids) must span at least two introns. Every retrogene found this way, was classified as 'KNOWN_PROTEIN_CODING'.

All of the retrocopies went through manual curation before the final release of the database. Genes that originated from transposons, and whose evolutionary conservation patterns seemed untrustworthy, were excluded. In addition, genes that provided a large number of retrocopies were carefully examined. In the comparison to the first release of the RetrogeneDB database [6], 316 human and 9 mouse retrocopies, which were located on chromosome patches, were also removed. This removal was dictated by the fact that retrocopies located on genomic patches are usually identical or extremely similar to their counterparts located on actual chromosomes or scaffolds. As a result, it is impossible to obtain unique mappings of reads (e.g., RNA-Seq, ChIP-Seq) to those regions.

**Homology**

The assignment of parental genes homology was done based on Ensembl 73 and Ensembl Plants 30 annotations for animals and plants, respectively. Detection of orthologous retrocopies was conducted within three separate species groups based on the Ensembl whole genome alignments for 13 *eutheria* species (*Homo sapiens, Pan troglodytes, Pongo abelii, Gorilla gorilla, Macaca mulatta, Callithrix jacchus, Mus musculus, Rattus norvegicus, Equus caballus, Sus scrofa, Bos taurus, Canis familiaris,* and *Oryctolagus cuniculus*), 5 *teleost* fish (*Tetraodon nigroviridis, Gasterosteus aculeatus, Takifugu rubripes, Oryzias latipes,* and *Danio rerio*) and 3 *neognath* birds (*Gallus gallus, Taeniopygia guttata,* and *Meleagris gallopavo*), as shown in supplementary figure SF1. Using the bx-python package (<https://github.com/bxlab/bx-python>), blocks of alignment containing retrocopies were extracted from the whole genome alignments. Retrocopy orthologous groups were then constructed based on their overlap within alignment blocks, which required at least 50% reciprocal overlap.

**Expression estimation based on RNA-Seq data**

To estimate the expression of both retrogenes and their parental genes, we utilized a unique hit approach, similar to the one used in RCPedia [7]. We downloaded short read libraries for various tissues from selected records of the NCBI SRA database and trimmed them using Trimmomatic [8]. Short reads matching known rRNA sequences were removed using Bowtie 2 [9]. Filtered reads were mapped to their reference genomes using STAR [10]. Reads that mapped uniquely to retrocopies and their parental genes were counted using the featureCounts program from the Subread package [11]. Expression estimates were normalized using the overall number of mapped reads, resulting in RPM (reads per million mapped reads) values.

**EST data analysis**

EST sequences of 43 species, listed in supplementary table S5, were downloaded from the UCSC database [12]. For a given species, all nucleotide sequences of retrocopies were aligned to the EST sequences using BLAST [13]. EST sequences, which had a sequence alignment less than 100 bp long and a coverage of less than 90%, were excluded from further analysis. All remaining EST sequences were then aligned to the reference genomes using BLAT [14]. An EST sequence was considered to be a retrocopy transcript if the EST – retrocopy alignment had an identity equal to or higher than 95% and EST coverage of at least 85%. Finally, EST alignment with a retrocopy had to have a higher score and identity than the alignment of the same EST with the parental gene or any other sequence.

**Polymerase activity annotation**

ChIP-Seq experiments provide valuable information of the exact DNA regions with which DNA-binding proteins interact. Taking advantage of the efforts made within the Encode Project [15], precomputed RNA Polymerase II Subunit A activity peaks from 53 human and 4 mouse ChIP-Seq libraries were used to annotate polymerase activity. All libraries are listed in supplementary table ST5. Mouse datasets were converted from the mm9 to the mm10 genome version using the liftOver tool from UCSC [12]. A polymerase peak was considered to be possibly linked to the retrocopy if its presence was detected within the annotated retrocopy body, down to 30% downstream of the 5’ end and no further than 1000 bp upstream of the 5’ end (supplementary figure SF2 A). Identification of such peaks was done using the intersect program from the BEDTools package [4]. At least 1 bp of overlap between the RNA Polymerase II peaks and the described region surrounding the 5’ end of the retrocopy was required.

For data visualization in the web browser, peaks from various libraries were merged using the merge program from the BEDTools package with the default settings. Additionally, for the visualization of the peak signal strength, the raw signal/coverage values were downloaded for the corresponding ChIP-Seq libraries in bigWig format. Their signal was then merged into one bigWig file per species, using the bigWigMerge program from UCSC [12].

**TSS annotations**

The FANTOM5 project has provided enormous insight into the alternative transcription start sites (TSS) landscape in human and mouse genomes, where the TSS positions were determined using the CAGE method and applied in a unified protocol to 1829 human and 1072 mouse libraries. This vast collection of libraries, covering *inter alia* tissues, cell lines and time-courses, was used for the annotation of retrocopies’ TSSs. The detailed description of all utilized libraries is provided in supplementary table ST6 [16]. FANTOM5 TSS annotations were downloaded from the official project website (<http://fantom.gsc.riken.jp/5/data/>) and converted into bed format. Mouse annotations were then converted from the mm9 to the mm10 genome version using the liftOver tool [12]. TSS was considered as a possible retrocopy alternative transcription start site if it was located near the 5’ ends of retrocopies, that is, in the region up to 500 bp upstream of the annotated retrocopy 5’ end, down to 30% of the annotated retrocopy body (supplementary figure SF2 B). The distance of 500 bp was adopted from the automated FANTOM5 protocols [17]. The transcription start sites potentially associated with retrocopies were identified using the intersect program from the BEDTools package [4], which requires an overlap of at least 1 bp between the annotated TSS positions and the described region near the 5’ ends of retrocopies. Each TSS, based on FANTOM project data, has specified expression level normalized to tags per million (TPM).

**References**

1.	Kielbasa, S.M., et al., *Adaptive seeds tame genomic sequence comparison.* Genome Res, 2011. **21**(3): p. 487-93.

2.	Flicek, P., et al., *Ensembl 2013.* Nucleic Acids Res, 2013. **41**(Database issue): p. D48-55.

3.	Kersey, P.J., et al., *Ensembl Genomes 2016: more genomes, more complexity.* Nucleic Acids Res, 2016. **44**(D1): p. D574-80.

4.	Quinlan, A.R. and I.M. Hall, *BEDTools: a flexible suite of utilities for comparing genomic features.* Bioinformatics, 2010. **26**(6): p. 841-2.

5.	Marques, A.C., et al., *Emergence of young human genes after a burst of retroposition in primates.* PLoS Biol, 2005. **3**(11): p. e357.

6.	Kabza, M., J. Ciomborowska, and I. Makalowska, *RetrogeneDB--a database of animal retrogenes.* Mol Biol Evol, 2014. **31**(7): p. 1646-8.

7.	Navarro, F.C. and P.A. Galante, *RCPedia: a database of retrocopied genes.* Bioinformatics, 2013. **29**(9): p. 1235-7.

8.	Bolger, A.M., M. Lohse, and B. Usadel, *Trimmomatic: a flexible trimmer for Illumina sequence data.* Bioinformatics, 2014. **30**(15): p. 2114-20.

9.	Langmead, B. and S.L. Salzberg, *Fast gapped-read alignment with Bowtie 2.* Nat Methods, 2012. **9**(4): p. 357-9.

10.	Dobin, A., et al., *STAR: ultrafast universal RNA-seq aligner.* Bioinformatics, 2013. **29**(1): p. 15-21.

11.	Liao, Y., G.K. Smyth, and W. Shi, *The Subread aligner: fast, accurate and scalable read mapping by seed-and-vote.* Nucleic Acids Res, 2013. **41**(10): p. e108.

12.	Hinrichs, A.S., et al., *The UCSC Genome Browser Database: update 2006.* Nucleic Acids Res, 2006. **34**(Database issue): p. D590-8.

13.	Altschul, S.F., et al., *Basic local alignment search tool.* J Mol Biol, 1990. **215**(3): p. 403-10.

14.	Kent, W.J., *BLAT--the BLAST-like alignment tool.* Genome Res, 2002. **12**(4): p. 656-64.

15.	Consortium, E.P., *An integrated encyclopedia of DNA elements in the human genome.* Nature, 2012. **489**(7414): p. 57-74.

16.	Lizio, M., et al., *Gateways to the FANTOM5 promoter level mammalian expression atlas.* Genome Biol, 2015. **16**: p. 22.

17.	Itoh, M., et al., *Automated workflow for preparation of cDNA for cap analysis of gene expression on a single molecule sequencer.* PLoS One, 2012. **7**(1): p. e30809.
